# HABITS AND ATTITUDES OF MOTHERS OF INFANTS IN RELATION TO BREASTFEEDING AND ARTIFICIAL FEEDING IN 11 BRAZILIAN CITIES

**DOI:** 10.1590/1984-0462/;2017;35;1;00014

**Published:** 2017

**Authors:** Mauro Batista de Morais, Ary Lopes Cardoso, Tamara Lazarini, Elaine Martins Bento Mosquera, Márcia Carvalho Mallozi

**Affiliations:** a Universidade Federal de São Paulo (Unifesp), São Paulo, SP, Brasil.; b Instituto da Criança da Faculdade de Medicina da Universidade de São Paulo (USP), São Paulo, SP, Brasil.; c Danone Early Life, Holanda.; d Danone Early Life Nutrition, São Paulo, SP, Brasil.; e Disciplina de Alergia, Imunologia e Reumatologia Pediátrica da Escola Paulista de Medicina, Unifesp, São Paulo, SP, Brasil.

**Keywords:** Breast feeding, Infant formula, Dried whole milk, Infant, Artificial feeding, Weaning

## Abstract

**Objective:**

To analyze the relationship between habits and attitudes of mothers and the types of milk offered to their children in their first two years of life.

**Methods:**

Retrospective study including 773 interviews of mothers from 11 Brazilian cities with children under 2 years of age. Interviews were conducted in 11 cities of Brazil. The following factors were analyzed: breastfeeding method planned during pregnancy and the method actually applied after birth; type(s) of milk(s) used on the day of the interview and earlier; age at which the child was introduced to whole milk; and source of advice used to choose a certain type of milk.

**Results:**

Breast milk was offered to 81.7% of infants during their first six months of life, to 52.2% of infants during their second semester (p<0.001) and to 32.9% of infants during their second year of life (p<0.001). In contrast, cow’s milk consumption increased from 31.1 to 83.8% (p<0.001) and 98.7% (p=0.05), respectively, for these three age groups. Infant (15.0%) and follow-on (also known as toddler’s) (2.3%) formulas were used by a much smaller number of infants than whole cow’s milk. Most mothers were not prescribed whole cow’s milk. Pediatricians were the health care professionals who most often recommended infant formulas.

**Conclusions:**

Rates of breastfeeding in Brazil remain below recommended levels. Brazilian mothers often decide to feed their infants with whole cow’s milk on their own initiative. The use of infant formulas after weaning is still too low.

## RESUMO

**Objetivo:**

Analisar a relação entre hábitos e atitudes de mães com os tipos de leite oferecidos para seus filhos nos dois primeiros anos de vida.

**Métodos:**

Estudo retrospectivo incluindo 773 entrevistas de mães de 11 cidades brasileiras com filhos com até 2 anos de idade. Foram analisadas as seguintes informações: tipo de aleitamento que planejava enquanto estava na gestação e o efetivamente realizado após o nascimento; tipo(s) de leite(s) utilizado(s) no dia da entrevista e anteriormente; idade de introdução do leite de vaca integral; e origem das recomendações para usar determinado tipo de leite.

**Resultados:**

O leite materno era oferecido para 81,7% dos lactentes no primeiro semestre de vida, 52,2% no segundo semestre (*p*<0,001) e 32,9% no segundo ano de vida (*p*<0,001). Por sua vez, o consumo de leite de vaca integral aumentou de 31,1 para 83,8% (*p*<0,001) e 98,7% (*p*=0,05), respectivamente, nestas três faixas etárias. Fórmula de partida (15,0%) e de seguimento (2,3%) eram utilizadas por um número de lactentes muito menor em relação aos que recebiam leite de vaca integral. A maioria das mães não recebeu prescrição de leite de vaca integral. Os pediatras foram os profissionais da área da saúde que mais frequentemente recomendaram fórmula infantil.

**Conclusão:**

As taxas de aleitamento natural no Brasil continuam abaixo das recomendações. As mães brasileiras, com frequência, decidem oferecer leite de vaca integral por iniciativa própria. É muito baixa a utilização de fórmula infantil quando o aleitamento natural é interrompido.

## INTRODUCTION

The first two years of life represent a critical period of growth and development for children. In the first six months, exclusive natural breastfeeding is ideal. Natural breastfeeding is recommended until two years of age, with complementary feeding starting at the age of six months.^1^ ^,^ ^2^ ^,^ ^3^ ^,^ ^4^ ^,^ ^5^

In Brazil, actions aimed at encouraging exclusive natural breastfeeding were improved during the early 1980s. A cross-sectional study conducted during the 2008 mass vaccination campaign, involving 34,366 infants living in Brazilian state capitals, showed that 41.0% of children were exclusively fed by natural breastfeeding during the first six months of life and that 59.0% of children were breastfed between 9 and 12 months of age. Another 1999 research study detected an increase in the median duration of exclusive natural breastfeeding from 23 to 54 days, as well as an increase in the median total breastfeeding duration from 296 to 342 days.^6^ In 2005, the mothers of 179 infants who did not receive exclusive natural breastfeeding reported that the feeding practices adopted were based on the experience of the mother herself and/or her family. Pediatrician recommendations and media information were respectively the second and third most common sources of information about breastfeeding.^7^ Only 12.0% of infants under 6 months of age who were not breastfed received infant formula as a substitute to breast milk, while that number was 7.0% for infants over 6 months of age. Most infants were fed whole cow’s milk. The median age at which infants were introduced to artificial feeding (i.e. bottle feeding) was three months,^7^ which was often performed inappropriately,^7^ confirming other Brazilian studies^8^ ^,^ ^9^ ^,^ ^10^. In addition to insufficient breastfeeding time duration, it was found that infant formulas are not being used as substitutes for breastfeeding often enough, contrary to Brazilian Pediatric Society recomendations.^1^ The Brazilian Ministry of Health^2^ warns that whole cow’s milk should be avoided during the infant’s first year of life, as it is poor in iron and may lead to overweight and further complications.

In this context, there is an increasing concern about the relationship between food and early life nutrition and the future development of chronic diseases such as diabetes mellitus, hypertension, and others.^1^ ^,^ ^11^ ^,^ ^12^ ^,^ ^13^ ^,^ ^14^ A systematic review showed that long-term breastfeeding was associated with lower blood pressure, total cholesterol, prevalence of overweight, type 2 diabetes, and with having better intellectual development.^11^

In 2009, *Danone Early Nutrition* conducted market research in Brazil on feeding practices during the first years of life, akin to what is practiced in other countries since 2003. Upon the release of the survey results, the authors of this article argued that a pediatric analysis of its data would be invaluable for providing input that could be used in campaigns to encourage healthier feeding practices during infancy.

Considering that in Brazil there is very limited information on nutrition during the first two years of life and its importance for present and future health, this study was conducted in order to analyze the relationship between dietary habits, mothers’ attitudes and the types of milk offered to their children in their first two years of life.

## METHOD

This retrospective study was carried out using information from a research database which was conducted with 773 mothers of children aged under 24 months living in 11 cities of the Northeast, Southeast, and South of Brazil. The research was based on a stratified convenience sample. The fieldwork occurred between February 2009 and February 2010. Information was collected in interviews conducted in the participants’ residences. The Synovate Research Institute (Rio de Janeiro, Brazil) was responsible for executing the field research and preparing the database.

As recruitment criteria, we selected mothers proportionally to the number of boys and girls and to the number of first children at each participating city. The 773 mothers recruited resided in: São Paulo (n=193), Rio de Janeiro (n=192), Campinas (n=32), Ribeirão Preto (n=37), Santos (n=33), São José dos Campos (n=30), Salvador (n=44), Recife (n=44), Fortaleza (n=44), Curitiba (n=63), and Porto Alegre (n=63). According to definitions set by the *Associação Brasileira de Empresas de Pesquisa* (ABEP),^15^ 53 (6.0%) respondents belonged to socioeconomic class A, 283 (36.6%) respondents belonged to class B, 356 (46.0%) respondents belonged to class C, 81 (10.5%) respondents belonged to class D, and none belonged to class E.^13^

Information collected during interviews was compiled into tables, including children’s age, birth order, maternal education, and insertion in the labor market. Interviews lasted approximately 50 minutes each. This study divided interview reports according to children’s age - first semester, second semester and second year of life.

The following interview questions were used in this study:

1. Before your child was born, what feeding method did you intend to use during their newborn period?
2. After childbirth, which feeding method did you actually use?
3. Which type(s) of milk(s) do you currently feed your child?
4. Which type(s) of milk(s) have you already fed your child?
5. At what age did your child began drinking whole cow’s milk (in liquid or powder form)?
6. Who recommended the feeding method(s) or type(s) of milk(s) that you currently use?

Statistical analysis was carried out in the Stacalc module of the EpiInfo software (Center of Disease Control and Prevention - CDC, Atlanta, USA) for calculating chi-squared test values.

This project was reviewed and approved by the Research Ethics Committee of the *Universidade Federal de São Paulo*, São Paulo Hospital (CEP 1006/11).

## RESULTS

Table 1 presents the sex, birth order, type of delivery, preterm birth, maternal age, and marital status according to age. Statistical analysis showed no statistically significant difference for any of these variables, with the exception of mothers under 20 years of age, who breastfed less frequently during their infants’ second year of life.

**Table 1:**
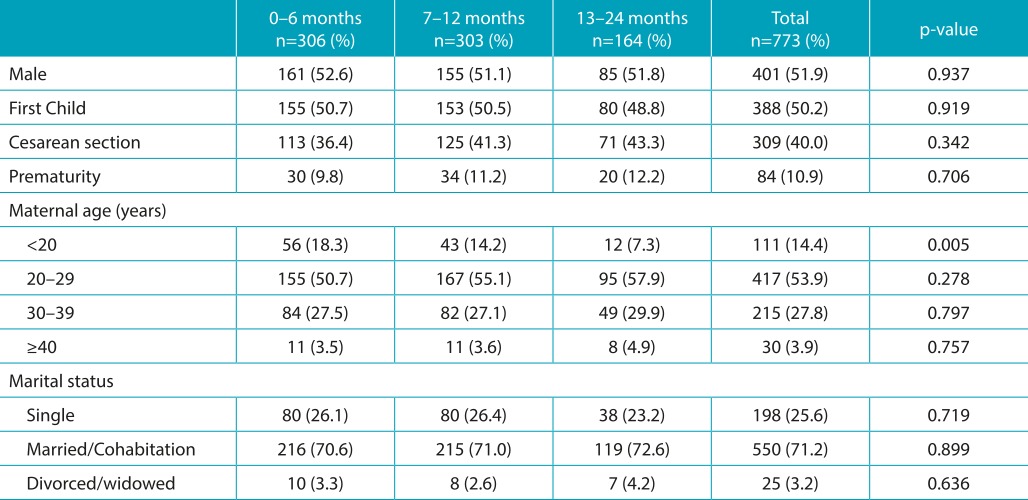
Sex, birth order, type of delivery, prematurity, maternal age, and marital status, according to age group.

The proportion of mothers under 20 years of age with children aged between 12 and 24 months was lower than the proportion of mothers under 20 years with children between 0 and 6 months of age (chi-square: *p*=0.002) and between 6 and 12 months of age (chi-square: *p*=0.040).

Among mothers of infants up to 6 months of age, 94.8% (290/306) had intended to breastfeed their children before birth. That number was 90.6% (275/303) for mothers of infants aged between 7 and 12 months, and 93.3% (153/164, p=0.152) for mothers of children aged between 13 and 24 months. In reality, breast milk was used soon after birth by 298 (97.4%) of the 306 mothers of infants under 6 months of age, by 290 (95.7%) of mothers with children aged between 7 and 12 months, and by 158 (96.3%) of those with children aged between 13 and 24 months (*p*=0.525).

Table 2 shows the types of milk fed to children at the time of the survey and those they had already been fed since birth. A portion of the infants was observed to have received more than one type of milk at the time of the survey. Among the infants, 81.7% of them received natural feeding in the first six months of life, a percentage that decreased progressively in the next six months and in the second year of life. The chi-squared test showed a statistically significant difference between the first and second semesters of life (*p*<0.001) and between the second semester and the second year of life (*p*<0.001). There was a statistically significant increase in the consumption of whole cow’s milk, which rose from 31.1% in the first semester of life to 83.8% (*p*<0.001) in the second semester, and to 98.7% (*p*=0.05) during the infants’ second year of life. The percentage of infants who received infant formula during their first six months of life (15.0%) was lower than that of those who received whole cow’s milk (31.1%). In the second semester of life, formula use (12.5%) was much lower than the use of whole cow’s milk (83.8%). Almost all infants (97.1%; or 751/773) had been breastfed at some period since birth. Considering only the children in the second semester and second year of life, it was determined that 41.9% and 37.8%, respectively, received infant formula. In contrast, whole cow’s milk consumption had already been administered to 34.6% of infants under 6 months of age and to more than 94.0% of infants over 6 months of age.

**Table 2:**
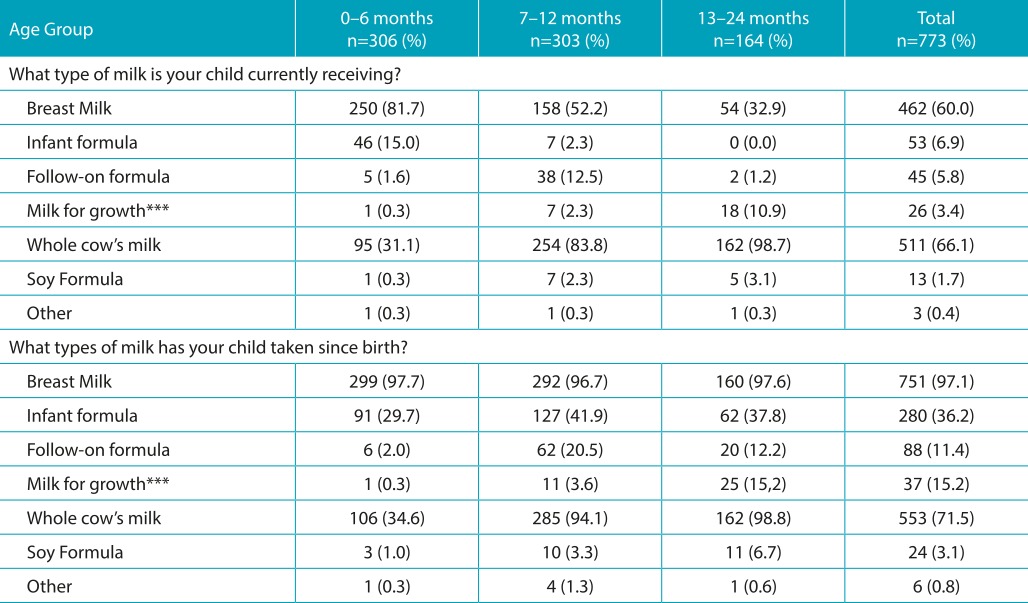
Types of milk used at the time of the survey* and types of milk used since the birth**.

*It is possible each child was fed more than one type of milk; **Each child could have received one or more types of milk since birth. ***Milk for growth: (definition) - milk adapted for children over 1 year of age.

Table 3 shows the age at which infants were introduced to whole cow’s milk among the three age groups. Considering the total of those groups, a monthly progressive increase in the number of infants that began a diet of whole cow’s milk during the first and fifth months of life was observed. However, it was also determined that in the sixth month there is a major increase in introducing whole cow’s milk in the diet - approximately a quarter of all infants. Introduction to cow’s milk between 5.1 and 6.0 months (for children between 6 and 24 months of age, 5.4%; 33/609) was lower than introduction between 6.1 and 7.0 months (25.9%, 121/467), which represents a statistically significant difference (*p*<0.001).

**Table 3:**
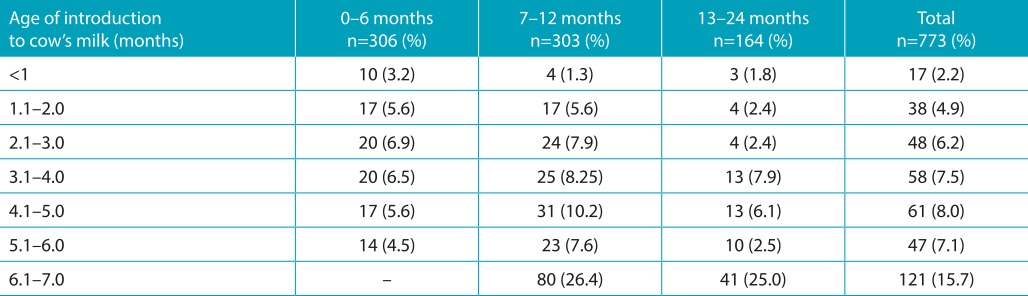
Age of introduction to cow’s milk in liquid or powder form, according to age group.

In Table 4, 54.9% of mothers of infants fewer than 6 months of age reported that they had not been instructed to use whole cow’s milk. 9.8% and 14.7% of the respondents mentioned advice from grandmothers and other family members, respectively. Pediatrician advice was mentioned in 13.7% of cases of children under 6 months, 18.6% for those in their second semester, and 27.6% for children over one year of age.

**Table 4:**
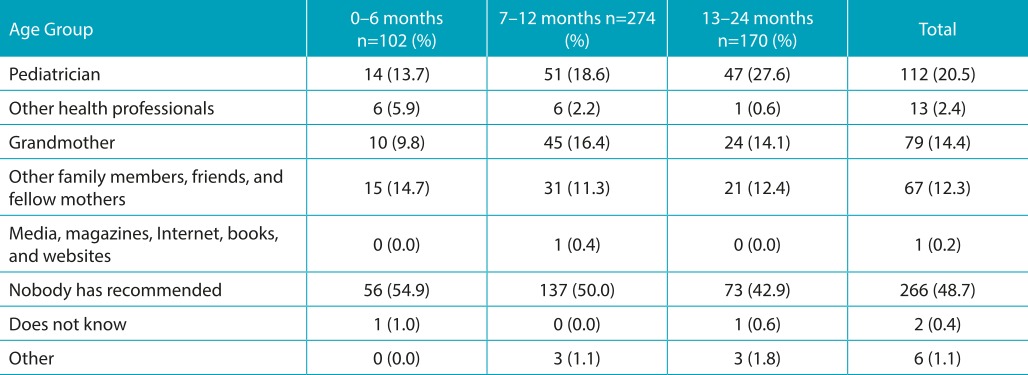
Person responsible for recommending* introduction to cow’s milk according to mothers of children of each of the age groups studied.

*For each respondent, more than one source of advice may have been mentioned.

According to the mothers interviewed, infant formula had been recommended only in the first and second semesters of life - 48% and 9% of respondents reported this, respectively. Out of the 48 nutritional types of advice in the first six months of life, 32 came from pediatricians, 2 from other health professionals, 3 from grandmothers, 4 from other family members/friends/fellow mothers, and 7 mothers received no type of advice whatsoever.

## DISCUSSION

The interviews showed that nearly all mothers during pregnancy had planned to breastfeed and indeed offered breast milk immediately after delivery. However, over the first six months of life, some infants began receiving other types of milk, especially whole cow’s milk. During the first year of life, infant formula was fed to approximately 15.0% of infants who were not being breastfed. From the sixth month onwards, an increase in the probability of introduction to cow’s milk was detected. The choice of the type of milk was frequently made by the mother and the pediatrician was the health care professional that most often made recommendations for formula use.

According to the Brazilian Pediatric Society, the Brazilian Ministry of Health, and the World Health Organization (WHO), exclusive natural breastfeeding should occur for at least six months.^1^ ^,^ ^2^ ^,^ ^3^ In this study, 81.7% of infants in the first semester of life received their mother’s milk. However, the information on the percentage of infants who exclusively were breastfed was not available. In order to calculate an estimate of the percentage of exclusively breastfed infants under 6 months of age, it was considered that if an infant receives two types of milk in this age group, it is most likely a case of mixed feeding. As shown in Table 2, 250 infants under six months of age were breastfed and 149 received other types of milk. Therefore, 93 infants received more than one type of milk (presumably breast milk and another type of milk, according to the current premise). Therefore, 157 (250 minus 93) were exclusively breastfed, yielding an estimated rate of exclusive breastfeeding of 51.3% (157/306). According to a 2008 vaccination campaign survey conducted in Brazilian state capitals and in the Federal District, 41.0% of infants were exclusively breastfed in the first 6 months of life,^6^ a number lower than the 51.3% estimate observed in this article. However, breastfeeding rates (81.7%) were similar to data obtained in a 2004 epidemiological survey conducted in the state of São Paulo (80.3%).^16^ In this study, 52.2% of infants in the second semester of life received both breast milk and other types of milk or food. This value can be considered similar to the 50.0% observed in São Paulo in 2004^16^ and the 58.7% detected for children between 9 and 12 months of age during the 2008 vaccination campaign.^6^ This data, related to the first year of infancy, unambiguously shows that breastfeeding rates in Brazil are still far below recommendations despite being on the rise since the 1980s.^17^

Considering this scenario, it is very important to encourage increased breastfeeding. It is essential to consider the determining factors for weaning in Brazil when developing campaigns and actions encouraging breastfeeding. The main factors associated with early weaning are: inadequate knowledge about the advantages and ideal duration of breastfeeding,^18^ the lack of participation of pregnant women and nursing mothers in programs encouraging breastfeeding,^19^ mothers with low levels of education, ^20^ primiparity and lack of experience with breastfeeding,^21^ ^,^ ^22^ ^,^ ^23^ difficulty to initiate breastfeeding immediately after birth,^21^ cleft nipple,^24^ mother’s conditions requiring use of medications, ^20^ pacifier use,^19^ ^,^ ^20^ ^,^ ^23^ ^,^ ^24^ ^,^ ^25^ and work.^22^ ^,^ ^24^ ^,^ ^25^ Therefore, educational measures must be targeted at specific audiences to increase the rate and duration of breastfeeding.

All research indicates that whole cow’s milk is the most common replacement for breast milk. In this study, cow’s milk was used by 31.1% of infants in the first six months of life, by 83.8% of infants in the second semester of life and by 98.7% of infants in the second year of life (Table 2). Approximately half of the mothers interviewed in all three age groups stated that no one had recommended whole cow’s milk for their children, that is, choice was made based on their own judgement. In 26.7% of cases, grandmothers, other family members, and friends were mentioned as sources of advice. According to 16.7% of mothers with children under 6 months, a pediatrician had recommended whole cow’s milk. That number rose to 18.6% for mothers of children in their second semester and 27.6% in the second year of life. The first six months were observed as the period during which whole milk usage increased the most. These findings indicate that an educational campaign should be organized to instruct mothers on the negative impact of feeding infants with whole cow’s milk and on the importance of breastfeeding until children complete 24 months of age, with complementary feeding after the first 6 months of life. It should be noted that, out of the 149 infants who in their first 6 months were not fed breast milk, only 46 (30.8%) were fed infant formulas, whereas 63.8% (95/149) were fed whole milk. For children in their second semester of life, these values were 12.1% (38/314) and 80.1% (254/314), respectively. These results clearly demonstrate that whole milk is used in lieu of infant formulas as a replacement for breast milk, contrary to recommendations by pediatric societies in Brazil^1^ and worldwide^4^ ^,^ ^5^ as well as the Ministry of Health,^2^ all of which claim whole - unmodified - cow’s milk should be avoided in the first year of infancy.

The results of this study confirm what was previously observed in São Paulo, Curitiba, and Recife,^7^ that is, infant formulas are used by a small number of infants, who cease to be naturally fed. Inadequate preparation of bottles by dilution or addition of sugar, cereals and chocolate milk powder^7^ was also observed. This creates negative repercussions on nutritional composition.^8^ ^,^ ^9^ ^,^ ^10^ There are many disadvantages of whole cow’s milk in comparison with infant formulas, including the former’s low essential fatty acid content; lower lactose content, which often motivates the addition of sucrose with its high cariogenic potential; excessive amount of proteins, which cause renal overload and increased risk of obesity in the future; and insufficient amount of iron with low bioavailability, an important risk factor for iron deficiency and iron deficiency anemia.^1^ The use of infant formula has been associated with a lower risk of iron deficiency anemia in infants between 7 and 12 months of age, even in comparison with those who received breast milk and other foods.^26^

In this retrospective study, a limiting factor could be the lack of sample size estimation. However, the number of interviews was sufficient to provide statistically significant differences in almost all analyses performed, indicating that the number of interviews was sufficient for characterizing correlations among variables. Another relevant aspect refers to the proportion of individuals in the sample studied belonging to upper socioeconomic classes, which is slightly higher than the overall Brazilian population. This factor must be taken into account when comparing this study’s results to the Brazilian population as a whole.

In conclusion, breastfeeding rates in Brazil remain below national and international recommendations. Brazilian mothers often decide on their own initiative to offer whole cow’s milk to their children in either the first or the second semesters of life. Despite their proven benefits, infant formulas are used by a small number of infants. A greater number of educational and incentive measures should be implemented to reverse such an unfavorable scenario for Brazilian infants.
